# Immune to Situation: The Self-Serving Bias in Unambiguous Contexts

**DOI:** 10.3389/fpsyg.2017.00822

**Published:** 2017-05-22

**Authors:** Xiaoyan Wang, Li Zheng, Lin Li, Yijie Zheng, Peng Sun, Fanzhi A. Zhou, Xiuyan Guo

**Affiliations:** ^1^School of Psychology and Cognitive Science, East China Normal UniversityShanghai, China; ^2^Shanghai Key Laboratory of Magnetic Resonance, Department of Physics, East China Normal UniversityShanghai, China; ^3^Key Laboratory of Brain Functional Genomics, Ministry of Education, Shanghai Key Laboratory of Brain Functional Genomics, School of Psychology and Cognitive Science, East China Normal UniversityShanghai, China; ^4^Zhejiang Yuying Education GroupHangzhou, China; ^5^Shanghai Key Laboratory of Magnetic Resonance, School of Psychology and Cognitive Science, East China Normal UniversityShanghai, China

**Keywords:** self-serving bias, self-enhancement, self-assessment, self-awareness, implicit causality

## Abstract

Traditionally, the self-serving bias has been investigated in ambiguous contexts in which participants work on tasks that measure novel abilities before making attributions without clear criteria for success or failure feedback. Prior studies have confirmed that the self-serving bias is pervasive in the general population, yet it varies significantly across situations involving ambiguous contexts. The present study features an unambiguous context encompassing interpersonal events that involved implicit causality (with the “self” as an actor or recipient), the inherent logic of which indicated attribution criteria. The aim of this study was to explore whether there is a self-serving bias in unambiguous contexts and to examine whether it is as sensitive to situation as it has been shown to be in ambiguous contexts. The results showed that, in an unambiguous context, participants exhibited self-serving bias in relation to attribution associated with negative interpersonal events. Additionally, the self-serving bias was greater in the actor condition relative to the recipient condition (Study 1), and this effect was not affected by the level of self-awareness, which was manipulated by the use or otherwise of a camera during the experiment (Study 2). Our findings provide evidence for the existence of the self-serving bias in unambiguous contexts. Moreover, the self-serving bias was shown to be immune to situation in unambiguous contexts, but it did depend on factors associated with the events *per se*, such as the actor versus recipient role that the self played in interpersonal events.

## Introduction

It is thought to be a fundamental need of individuals to try to maintain positive beliefs about themselves (Heine et al., 1999; Mezulis et al., 2004; Sedikides and Alicke, 2012). These positive beliefs about the self can be manifested through what is known as the “self-serving bias,” which refers to individuals taking responsibility for success but blaming others for failure (Miller and Ross, 1975; Miller, 1976, 1978; Larson, 1977; Bradley, 1978, 1979; Sedikides et al., 1998; Duval and Silvia, 2002; Mezulis et al., 2004). Moreover, a self-serving bias is considered by many researchers to be essential for an individual’s mental health and adaptive functions (Taylor and Brown, 1994; Heine et al., 1999; Mezulis et al., 2004; Sedikides and Alicke, 2012).

In previous studies on the self-serving bias, participants were often asked to work on a task, and then were given random “success” or “failure” outcome feedbacks (Larson, 1977; Urban and Witt, 1990; Sedikides et al., 1998). In most cases, tasks measuring purportedly novel abilities or characteristics were adopted to make the outcome feedbacks more plausible in these studies (Larson, 1977; Sedikides et al., 1998; Duval and Silvia, 2002). As a result, individuals had no clear clues or objective criteria with which to ascertain responsibility attribution for the outcome. For this reason, the attributional context can be seen as being ambiguous in these previous studies. Indeed, for many years, the self-serving bias has been investigated in such ambiguous contexts. Studies have confirmed that the self-serving bias is pervasive in the general population but that it demonstrates significant variability across age, culture, and situation (Sedikides et al., 1998; Duval and Silvia, 2002; Mezulis et al., 2004; Coleman, 2011; Colonnello and Heinrichs, 2014). Other studies’ findings have suggested that individuals manifest a self-serving bias because they wish to enhance or protect their self-esteem, which has been identified as a “self-enhancement” or “self-protection” motivation (Bradley, 1978; Cunningham et al., 1979; Sedikides et al., 1998). Such motivations have been shown to engender a preference for fostering a positive self-concept (Duval and Silvia, 2002). In addition, it has been posited that people possess a “self-assessment” motivation to seek accurate information about the extent of their abilities and the correctness of their opinions, which can occasionally conflict with one’s self-enhancement/self-protection motivations (Sedikides, 1993; Sedikides and Strube, 1997; Duval and Silvia, 2002). When there are few objective criteria with which to evaluate the correctness of attribution in an ambiguous context, self-assessment concerns are reduced, thus, conflict between self-enhancement/self-protection and self-assessment motivations tends to be weaker in ambiguous contexts.

Importantly, the self-serving bias can also be measured and manifested in an unambiguous context. At many moments in life, the inherent logic of interpersonal events can give people clues and criteria that they can use to attribute these events to a particular cause or causes, rendering the attributional context unambiguous. Considering the following two descriptions featuring “implicit causality verbs,” (i.e., verbs that carry important implications with regard to which person is perceived as being causally responsible for the described event) (Garvey and Caramazza, 1974; Rudolph and Fõrsterling, 1997): “Mary hits Lisa” and “Mary protects Lisa.” Because of the causality implications of the verbs in these descriptions, most people will tend to attribute the former event to “Mary” (i.e., the actor of the event, and the subject of the sentence) and the latter to “Lisa” (i.e., the recipient of the event, and the object of the sentence) (Caramazza et al., 1977; Rudolph and Fõrsterling, 1997). The present study features implicit causality events and substitutes “self” for the actor or recipient of the interpersonal event (e.g., “I hit YangLi” or “WangShan hits me”), in order that the self-serving bias could be examined in an unambiguous context. As the implicit causality of interpersonal events may provide attributional criteria, an individual’s self-assessment motivation might activate intensively, and, accordingly, there may be intensive conflicts between the self-enhancement/self-protection and the self-assessment motivations in an unambiguous context. In the present study, we aim to investigate whether the self-serving bias can be sufficiently robust to survive in an unambiguous context, such as people commonly experience in real life. Moreover, the unambiguous context provides a chance to set a conflict condition in which attributional clues and criteria may restrain an individual’s self-enhancement/self-protection motivations, and to investigate the self-serving bias with respect to such conflicts. Studies have shown that one’s self-enhancement/self-protection motivations are dominant among many motives (Sedikides, 1993; Sedikides and Strube, 1997). Thus, we predict that there will be a self-serving bias in an unambiguous context.

Previous studies have demonstrated that some situational factors affect the self-serving bias in ambiguous contexts. For example, people’s self-serving biases have been found to be enhanced under the focus of a camera (Duval and Silvia, 2002), and researchers have suggested that self-awareness levels are elevated in this situation (Scheier and Carver, 1983; Govern and Marsch, 2001; Silvia and Duval, 2001; Duval and Silvia, 2002; Carver, 2012; Silvia and Phillips, 2013). Numerous studies have documented that high self-awareness can be induced through using a camera, a mirror, one’s own voice, mindfulness, the I-priming procedure, and self-face recognition (Berkowitz, 1987; Duval and Silvia, 2002; Brown and Ryan, 2003; Ma and Han, 2009, 2010; Wiekens and Stapel, 2010). Enhanced self-awareness beyond baseline levels can increase people’s positive emotional states and their willingness to help (Berkowitz, 1987; Brown and Ryan, 2003). In addition, researchers have argued that an individual can be more aware of the current state of the self with increasing self-awareness, and discriminate the current state from the ideal standard more easily. This discrimination might threaten one’s self-esteem, and in turn led to increased motivation to enhance or protect it (Duval and Lalwani, 1999; Duval and Silvia, 2002). Previous studies have discovered that, when making attributions in ambiguous context, one’s self-serving bias may be vulnerable to be influenced by the level of self-awareness (Silvia and Duval, 2001; Duval and Silvia, 2002). A question remians unanswered of whether a self-serving bias in an unambiguous context is altered by the level of self-awareness operating in a similar manner as in ambiguous context. In the present study, our second goal is to test the impact of a camera on the self-serving bias in an unambiguous context. Numerous studies have claimed that individuals in a conflict task are motivated to devote their cognitive resources to conflict solving and not to expend these precious resources on features irrelevant to the task itself (Botvinick, 2007; Dignath et al., 2015). In an unambiguous context, the inherent logic of the interpersonal events could provide people with clues and criteria that they can use to attribute these events to some cause or causes, activating their self-assessment motivation. People’s self-enhancement/self-protection motivations may conflict intensely with their self-assessment motivation, such that individuals might be motivated to devote cognitive resources to solving the conflict and bringing about self-harmony. In which case, one’s attribution would be barely any different across various situations in an unambiguous context. We anticipate that people’s self-serving bias will be hardly influenced by using a camera or not in an unambiguous attribution context.

To summarize, we conduct two studies to test our hypotheses. In Study 1, we examine the self-serving bias effect in an unambiguous context. Participants are presented with descriptions of self-relevant (wherein the “self” plays the role of an actor or recipient) and other-relevant implicit causality interpersonal events, and are asked to attribute the events to one of two interacting persons. The self-serving bias is measured through the difference between the probability of attribution to the self (self was an actor or recipient) and the probability of attribution to the other as actor or recipient for other-relevant events. In Study 2, we manipulate individual self-awareness levels by using or not using a video camera (Duval and Silvia, 2002) in order to examine its impact on the self-serving bias in an unambiguous context.

## Study 1

In Study 1, we examined the self-serving bias in an unambiguous context. Participants were exposed to an implicit causality disambiguation task (Caramazza et al., 1977; Blankenship and Craig, 2012) in which they were presented with descriptions of self-relevant and other-relevant implicit causality interpersonal events and were asked to attribute the events to one of the two interacting persons.

### Materials and Methods

#### Participants

Twenty-two right-handed volunteers from the university community with normal or corrected-to-normal vision participated in the study (of these, 12 were female, and all were between 19 and 23 years old, *M* = 20.6, *SD* = 0.82). Additionally, one participant was excluded because they provided insufficient button responses. All participants gave their informed consent before the test, and they were paid for their participation. This study was approved by the Ethical Committee of East China Normal University.

#### Materials and Design

Forty Chinese two-character implicit causality verbs—20 of which were positively valenced and 20 negatively valenced—were used in the present study. These verbs were selected from a pretest. Firstly, 162 implicit causality verbs were selected and translated from previous studies (Garvey and Caramazza, 1974; Kasof and Lee, 1993; Goikoetxea et al., 2008; Ferstl et al., 2011). Secondly, 30 participants who did not participate in the formal experiment were presented with 162 sentence fragments in the format “NP1 V NP2 because Pro…,” in which “NP1” denotes the first noun phrase, “NP2” the second noun phrase, “V” refers to the verb, and “Pro” to the pronoun (e.g., “YangLi hits WangShan, because she…”). Then, the participants were asked to give a reason or motive for the action. Their responses were codified by two independent raters, and all of the verbs were found to fall into three types: NP1-biased, NP2-biased, and Indeterminable (Garvey and Caramazza, 1974; Goikoetxea et al., 2008; Cozijn et al., 2011; Ferstl et al., 2011). “NP1-biased” means that the participants agreed in assigning the response to the question to the subject of sentence (NP1); “NP2-biased” denotes that participants agreed in assigning the pronoun to the object of the sentence (NP2); and “indeterminable” signifies that participants did not agree in assigning the pronoun, and neither NP1-biased or NP2-biased verb types predominated (Garvey and Caramazza, 1974). A one-way chi-square test for each verb was calculated separately, testing the null hypothesis of equal expected frequencies across the two categories of bias responses (Goikoetxea et al., 2008). Thus, 51 NP1-biased and 56 NP2-biased verbs were selected according the criteria mentioned above. Thirdly, the emotional valence of each of these implicit causality verbs was evaluated using a 9-point scale of unpleasant-pleasant ratings (1 = unpleasant, 9 = pleasant) from the Chinese Affective Words System (Wang et al., 2008). As a result, 20 Chinese two-character positively (a “pleasant” ratings of more than 6) and 20 negatively (a “pleasant” ratings of less than 3.5) valenced implicit causality verbs were selected. These two sets of verbs differed in valence [positive (*M* = 6.56, *SE* = 0.06), negative (*M* = 2.93, *SE* = 0.03), *t*(19) = 75.22, *p* < 0.001] but were alike for arousal [positive: *M* = 5.33, *SE* = 0.16, negative: *M* = 5.47, *SE* = 0.10, *t*(19) = 0.80, *p* = 0.43], familiarity [positive: *M* = 5.86, *SE* = 0.09, negative: *M* = 5.60, *SE* = 0.08, *t*(19) = 2.00, *p* = 0.06], and frequency [positive: *M* = 22.65, *SE* = 3.01, negative: *M* = 22.80, *SE* = 6.21, *t*(19) = 0.02, *p* = 0.98]. Within each set, 10 verbs were NP1-biased and the others were NP2-biased.

These implicit causality verbs were used to construct three kinds of one-sentence interpersonal events. Each sentence comprised one subject, one verb, and one object. For self-relevant events, “self” was assigned as an actor or as a recipient. In the actor condition, “self” was the subject of the sentence, and a Chinese proper name was selected as the object of the sentence (e.g., “I hit YangMing because ______ am/is that kind of person”). In the recipient condition, “self” was the object of the sentence, and a Chinese proper name was selected as the subject of the sentence (e.g., “CaoHua hits me because ______ am/is that kind of person”). For other-relevant events, sentences were written in the third person and contained common Chinese names; these names were randomly placed as the subject or the object of the sentence (e.g., “WangShan hits LiMin because ______ is that kind of person”).

For self-relevant positive and negative events, the probability of attribution to the self (self was an actor or recipient) was calculated. The probability of attribution to an actor or to a recipient for other-relevant events were calculated separately, as the corresponding baselines. The delta values of attribution probability (self–other) were used to evaluate the self-serving bias in specific conditions. The experimental design was a 2 (Role: actor or recipient) × 2 (Valence: positive or negative) within-subject design.

#### Procedure

Participants arrived individually and 120 sentences depicting interpersonal events were presented to them randomly via a computer screen. Participants were asked to read the sentence and to fill in the gap therein by selecting one of the two names in the sentence as quickly as possible. The positions of the two names were randomly assigned to the left or right side below the sentence. Participants were asked to press the “F” or “J” on the keyboard if they want to choose the left or right name, respectively. At the end of the process, participants were debriefed and thanked for their participation.

### Results and Discussion

#### Attribution Responses

To examine the self-serving bias, the delta values of attribution probability (self–other) across conditions were calculated. A one-sample *t*-test revealed that the delta values were significantly lower than zero in negative events [actor: *M* = -0.30, *SE* = 0.04, *t*(21) = -8.70, *p* < 0.001; recipient: *M* = -0.15, *SE* = 0.03, *t*(21) = -4.86, *p* < 0.001] but not in positive events [actor: *M* = -0.05, *SE* = 0.02, *t*(21) = -1.96, *p* = 0.06; recipient: *M* = -0.05, *SE* = 0.03, *t*(21) = -1.63, *p* = 0.14]. These results suggest that people manifest a self-serving bias in evaluating negative events but not in evaluating positive events.

**FIGURE 1:**
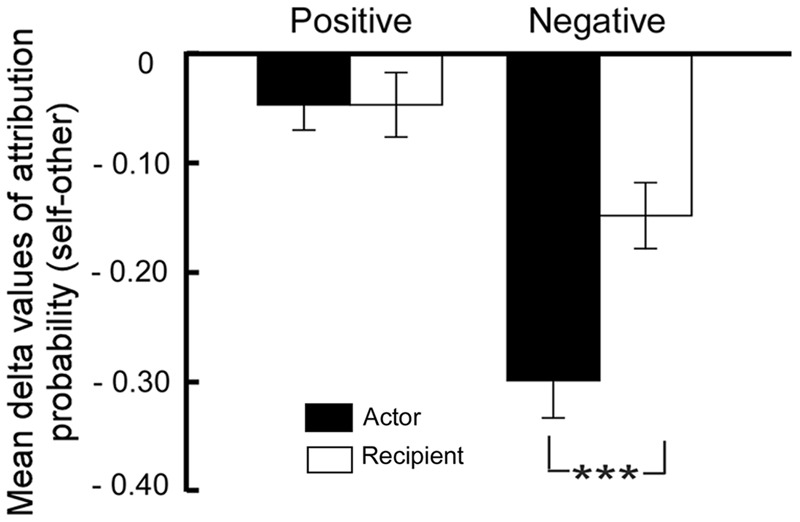
**Mean delta values (self–other) when the self was an actor or a recipient in positive and in negative interpersonal events (^∗∗∗^*p* < 0.001; error bars indicate standard error of the mean)**.

For the delta values across conditions, a 2 (Role: actor or recipient) × 2 (Valence: positive or negative) repeated measures analysis of variance (ANOVA) revealed the main effects of role [*F*(1,21) = 5.65, *p* = 0.03, $\eta_{p}^{2}$ = 0.21], and valence [*F*(1,21) = 27.53, *p* < 0.001, $\eta_{p}^{2}$ = 0.57]. The Role × Valence interaction was significant [*F*(1,21) = 13.78, *p* = 0.001, $\eta_{p}^{2}$ = 0.40]. See also **Figure 1**. Simple effect analysis revealed that there was greater self-serving bias in the actor relative to the recipient condition in negative events [*F*(1,21) = 21.54, *p* < 0.001, $\eta_{p}^{2}$ = 0.51] but not in positive events [*F*(1, 21) = 0, *p* = 1.0].

#### Reaction Times

Regarding the reaction times, participants’ attributional responses to the self (when self was an actor or recipient) in self-relevant events and their responses to other (when other was an actor or recipient) in these other-relevant events were calculated. A 2 (Target: self or other) × 2 (Role: actor or recipient) × 2 (Valence: positive or negative) repeated measures ANOVA revealed the main effects of target [*F*(1,21) = 41.35, *p* < 0.001, $\eta_{p}^{2}$ = 0.66] and valence [*F*(1,21) = 14.34, *p* = 0.001, $\eta_{p}^{2}$ = 0.41]. The Target × Valence interaction was significant [*F*(1,21) = 12.87, *p* = 0.002, $\eta_{p}^{2}$ = 0.38]. The Role × Valence interaction was also significant [*F*(1,21) = 8.23, *p* = 0.009, $\eta_{p}^{2}$ = 0.28]. There was no other significant main effect or interactions, all *F* < 3.61, all *p* > 0.07. Further simple effect analysis revealed that participants undertook faster responses for self-relevant positive events (*M* = 2995 ms, *SE* = 180) relative to negative events (*M* = 3775 ms, *SE* = 281) [*F*(1,21) = 22.41, *p* < 0.001, $\eta_{p}^{2}$ = 0.52]. There was no significant difference between their response times for other-relevant positive (*M* = 3999 ms, *SE* = 237) and negative events (*M* = 4118 ms, *SE* = 238) [*F*(1,21) = 0.79, *p* = 0.39]. Additionally, participants completed faster responses for evaluating negative events in the actor (*M* = 3733 ms, *SE* = 217) relative to the recipient (*M* = 4161 ms, *SE* = 299) condition [*F*(1,21) = 6.52, *p* = 0.02, $\eta_{p}^{2}$ = 0.24]. There was no significant difference between the actor (*M* = 3549 ms, *SE* = 213) and the recipient (*M* = 3446 ms, *SE* = 209) condition for evaluating positive events [*F*(1,21) = 0.75, *p* = 0.40].

These results suggest that participants are more likely to isolate the self from negative events, indicating there is a self-serving bias present in unambiguous contexts. Additionally, the self-serving bias in the present unambiguous context was greater when participants played the role of an actor relative to a recipient. Moreover, participants completed faster responses in the actor relative to the recipient condition for evaluating negative events, indicating that people may employ less cognitive resources to make attributions, and thus exhibit a relatively intuitive self-serving response in the actor condition.

## Study 2

In Study 2, we explored the effect of self-awareness on the attributional pattern in unambiguous contexts. Self-awareness levels were manipulated through the use or otherwise of a video camera during the experiment (Alden et al., 1992; Duval and Silvia, 2002). Attributional patterns were measured using the same procedures as were used in Study 1.

### Materials and Methods

#### Participants

The study’s participants were 48 volunteers from the university community with normal or corrected-to-normal vision (24 of whom were female, and all were between 17 and 28 years old, *M* = 19.9, *SD* = 1.84). Each participant was randomly assigned to either a high self-awareness group (25 volunteers) or a low self-awareness group (23 volunteers). All participants gave their informed consent before the study, and they were paid for their participation. This study was approved by the Ethical Committee of East China Normal University.

#### Procedure

Participants arrived individually and were each told that the study was part of a research program sponsored by the National Institute for the Study of Attribution. The procedure used was almost identical to that used in Study 1, except that self-awareness was manipulated during the experiment.

##### Self-awareness manipulation

In the high self-awareness group, a tripod-mounted video camera was placed 80 cm away from the participants. Each was told that the National Institute requested the videotaping of a random sample of subjects, presumably to ensure standardization of testing conditions, and that he/she had been randomly selected for videotaping. In fact, the video would be erased after they completed the experimental task. In the low self-awareness group, the video camera was turned off and faced the wall. Participants were given the same information but were told they had not been randomly chosen for videotaping.

Following this manipulation of their self-awareness, participants completed the same disambiguation task that was used in Study 1. At the end of the task, participants were probed for suspicion and debriefed.

### Results and Discussion

#### Attribution Responses

As in Study 1, the delta values (self–other) of attribution probability were calculated to examine the self-serving bias across conditions. A one-sample *t*-test revealed that, when self-awareness was high, the delta values were significant lower than zero in negative events [actor: *M* = -0.30, *SE* = 0.04, *t*(21) = -6.58, *p* < 0.001; recipient: *M* = -0.11, *SE* = 0.03, *t*(21) = -3.01, *p* < 0.001] but not in positive events [actor: *M* = -0.06, *SE* = 0.03, *t*(21) = -1.44, *p* = 0.16; recipient: *M* = -0.02, *SE* = 0.03, *t*(21) = -0.86, *p* = 0.40]. Similar results were observed when self-awareness was low. Thus, for positive events, actor (*M* = -0.02, *SE* = 0.03), *t*(21) = -0.74, *p* = 0.47; recipient (*M* = -0.02, *SE* = 0.03), *t*(21) = -0.52, *p* = 0.61. While, for negative events, actor (*M* = -0.28, *SE* = 0.04), *t*(21) = -7.07, *p* < 0.001; recipient (*M* = -0.16, *SE* = 0.04), *t*(21) = -5.49, *p* < 0.001. These results indicate that people manifest a self-serving bias in evaluating negative events but not in relation to positive events, irrespective of whether their self-awareness levels are high or low.

The 2 (Role: actor or recipient) × 2 (Valence: positive or negative) × 2 (Self-awareness: high or low) repeated measures ANOVA revealed the main effects of role [*F*(1,46) = 13.66, *p* = 0.001, $\eta_{p}^{2}$ = 0.23] and valence [*F*(1,46) = 41.97, *p* < 0.001, $\eta_{p}^{2}$ = 0.48]. The Role × Valence interaction was significant [*F*(1,46) = 9.60, *p* = 0.003, $\eta_{p}^{2}$ = 0.17]. There was no main effect of self-awareness or interactions between self-awareness and any other variables, all *F* < 1.37, all *p* > 0.25. Simple effect analysis revealed that there was greater self-serving bias in the actor relative to the recipient condition in negative events [*F*(1,47) = 19.56, *p* < 0.001, $\eta_{p}^{2}$ = 0.29] but not in positive events [*F*(1,47) = 0.42, *p* = 0.52].

#### Reaction Times

The 2 (Target: self or other) × 2 (Role: actor or recipient) × 2 (Valence: positive or negative) × 2 (Self-awareness: high or low) repeated measure ANOVA revealed the main effects of target [*F*(1,46) = 63.56, *p* < 0.001, $\eta_{p}^{2}$ = 0.58] and valence [*F*(1,46) = 5.31, *p* = 0.03, $\eta_{p}^{2}$ = 0.10]. There was no other significant main effect or interactions, all *F* < 3.08, all *p* > 0.09. Pairwise comparisons revealed that participants completed faster responses for positive events (*M* = 3145 ms, *SE* = 114) relative to negative events (*M* = 3329 ms, *SE* = 123), *p* = 0.03, and participants undertook faster responses for evaluating self-relevant (*M* = 2899 ms, *SE* = 103) relative to other-relevant events (*M* = 3575 ms, *SE* = 133), *p* < 0.001.

These results replicate the findings from Study 1, demonstrating that participants manifested a self-serving bias. Furthermore, the self-serving bias was found to be greater when participants played the role of an actor relative to that of a recipient. However, the appearance of the self-serving bias in this study’s unambiguous context was not affected by the use or otherwise of a camera.

## General Discussion

Our study explored the self-serving bias and the impact of self-awareness on it in unambiguous contexts. We found that participants exhibited a self-serving in relation to negative interpersonal events in an unambiguous context, and that it was greater when the self played the role of an actor compared to that of a recipient. Moreover, this attributional pattern was not affected by the inclusion of a camera in an unambiguous context, but rather was mainly dependent upon factors associated with the events *per se*, such as the actor or recipient role the self played in the implicit causality interpersonal events.

The self-serving bias had been expounded in connection with taking credit for success (internal attribution of positive events: the “self-enhancing” bias) and with denying responsibility for failure (external attribution for negative events: the “self-protective” bias) (Cunningham et al., 1979; Blackwood et al., 2003; Hepper and Sedikides, 2012). Our results suggest that, in an unambiguous context, people are more likely to isolate the self from negative events, and manifest only a self-protection bias. Previous studies have argued that striving to enhance a relationship with positive events serves self-enhancement, whereas endeavoring to avoid blame for negative events serves self-protection (Cunningham et al., 1979; Blackwood et al., 2003; Alicke and Sedikides, 2009; Hepper and Sedikides, 2012). Generally, self-enhancement regulates the superordinate need to view oneself positively by making slight adjustments in response to environmental disturbances. Self-protection is, by contrast, an emergency system that operates when self-image is threatened below a particular tolerance point (Alicke and Sedikides, 2009; Hepper and Sedikides, 2012). In an unambiguous context, attributional clues and criteria in implicit causality interpersonal events can cause one’s self-assessment motivation to become strongly activated, which promotes the seeking and favoring of information that provides accurate knowledge about the self, rather than flattering one’s self-concept (Sedikides and Strube, 1997; Duval and Silvia, 2002). As a result, people manifest a self-protection bias rather than a self-enhancement bias for the very reason that one’s self-protection motivation is more intensive. Alternatively, previous studies have revealed that people do not like those who manifest a self-enhancement bias (Hoorens, 2011). Individuals perceived the target as more immoral, unintelligent, and unfriendly when the target self-enhanced either intentionally or unintentionally, rather than self-presenting accurately (Lafrenière et al., 2016).

Our study also found that, in an unambiguous context, the self-serving bias is immune to situation: the participants’ self-serving bias was not affected by their level of self-awareness, which was manipulated by the use or otherwise of a camera in the specified situation. Numerous studies have claimed that individuals in a conflict task are motivated to devote their cognitive resources to the conflict solving and not to expend these precious resources on features irrelevant to the task itself (Botvinick, 2007; Dignath et al., 2015). We posit that, in an unambiguous context, people tend to isolate the self from negative events because of the self-protection motivation. Furthermore, they may also be likely to attribute events to a person recognized by the psychological causality implicit in the unambiguous context because of the self-assessment motivation. Thus, an individual’s self-protection motivation may conflict intensively with his/her self-assessment motivation in an unambiguous context. In such a conflict task, individuals might be motivated to devote cognitive resources to solving the conflict and in order to bring about self-harmony. A further point for consideration is that we did not manipulate the magnitude of the conflict in the unambiguous context. That is, what might happen if we were to reduce the conflicts between the self-enhancement/self-protection and self-assessment motivations in unambiguous contexts? Additionally, previous studies have shown that an individual’s self-serving bias is influenced by using or not using a camera when making attributions in an ambiguous context (Silvia and Duval, 2001; Duval and Silvia, 2002), yet we did not replicate the effect of self-awareness on the self-serving bias in our study’s unambiguous context. We expect that there may be different attributional processes between these two attributional contexts. In an ambiguous context, because there are no clear attributional criteria, self-assessment concerns are reduced, an individual’s self-enhancement/self-protection motivations may play an important role in attribution. Conversely, in an unambiguous context, because the attributional criteria are relatively clear, an individual’s self-assessment motivation may activate intensively, and attribution might depend on self-enhancement/self-protection and self-assessment motivations simultaneously. Moreover, people exhibited self-protection bias in our study, indicating that this bias is so intensive that it is not influenced by external criteria, such as the implicit causality information available in an unambiguous context. In addition, in previous studies in which participants were given negative feedback and were asked to make attributions in relation to the feedback, individuals manifested the self-serving bias based on their self-protection motivations, which was enhanced by their increased self-awareness (Silvia and Duval, 2001; Duval and Silvia, 2002). On the contrary, the self-protection bias was immune to the level of self-awareness present in our study, suggesting that it would not be heightened significantly. These results also point to the restraining effect of the self-assessment motivation in an unambiguous context. Although the effectiveness of a camera in enhancing self-awareness has been verified in previous studies (e.g., Silvia and Duval, 2001; Duval and Silvia, 2002), due to the lack of a manipulation check, the present study did not provide direct evidence that the camera enhanced self-awareness. Therefore, caution should be applied when interpreting these findings of the present study, and further research is needed to better understand the effect of self-awareness on the self-serving bias in an unambiguous context.

Noteworthily, of particular interest is our finding that the study’s participants manifested greater self-serving bias when the attributional target took the role of an actor relative to the role of a recipient in an unambiguous context. That is, the self-serving bias was modulated by factors associated with the event itself. In prior research, the self-serving bias has been considered as a heuristic judgment (Dunning et al., 1989; Chambers and Windschitl, 2004; Beer and Hughes, 2010) that is made more quickly and requires fewer cognitive resources than accurate self-evaluation (Beer and Hughes, 2010). In our study, finding a reduced self-serving bias in the recipient condition suggests that more complicated self-evaluation processes, which are more cognitively demanding than heuristic judgments, are involved in the judgments. This inference is generally consistent with those reported by Wang et al. (2015), whose neuroimaging results illustrated that dorsal medial prefrontal cortex engagement corresponding to self-evaluation shows greater activity when people take longer reaction time to make less self-serving evaluations in the recipient condition.

In conclusion, the present study provides evidence for the existence of the self-serving bias in unambiguous context. Allowing that people’s self-enhancement/self-protection and self-assessment motivations may conflict intensely, the self-serving bias was immune to a situation in an unambiguous context, and, instead, was mainly dependent upon factors associated with the events *per se*, such as the actor or recipient role the self played in the interpersonal events.

## Ethics Statement

This study was carried out in accordance with the recommendations of the Ethical Committee of East China Normal University with written informed consent from all subjects. All subjects gave written informed consent in accordance with the Declaration of Helsinki. The protocol was approved by the Ethical Committee of East China Normal University.

## Author Contributions

XW, LZ, LL, and XG devised the concept and supervised the study. XW and YZ collected the data. XW, LZ, LL, PS, and XG joined in the interpretation of data. XW, LZ, LL, FZ, and XG carried out the writing of the manuscript.

## Conflict of Interest Statement

The authors declare that the research was conducted in the absence of any commercial or financial relationships that could be construed as a potential conflict of interest.
